# Supplementary material for: Tany-Seq: Integrated Analysis of the Mouse Tanycyte Transcriptome
Source: Cells. 2022 May 6;11(9):1565. doi: 10.3390/cells11091565 (PMC9104898; doi:10.3390/cells11091565)
Supplement: Supplementary file 1 [file cells-11-01565-s001.zip › cells-1690609-supplementary.pdf]

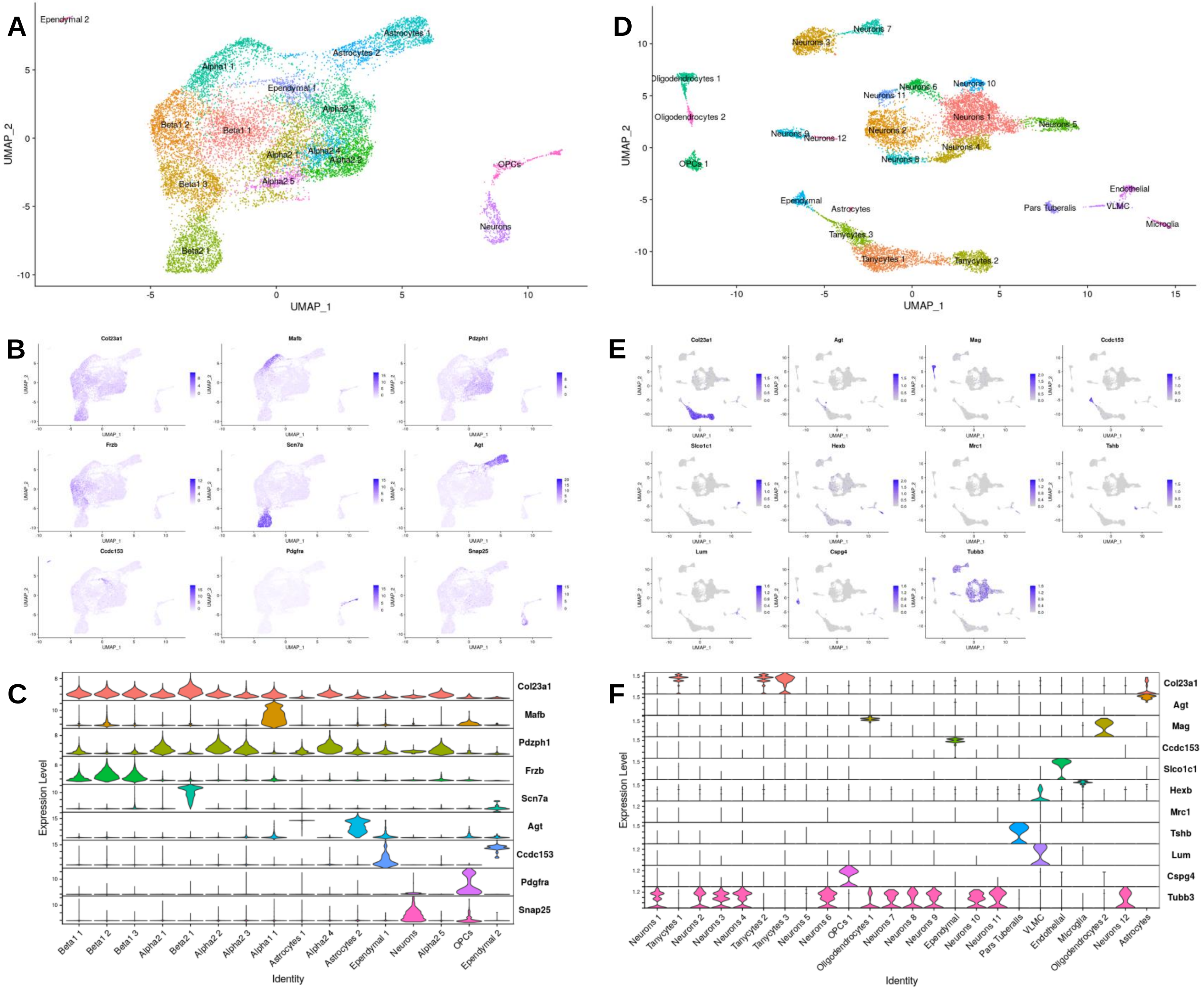

Figure S1: Identification of cell clusters in hypothalamic reference datasets. **(A)** UMAP of hypothalamic single cell RNA sequencing dataset. Raw data by Yoo et al. **(B)** Featureplots of cell-type specific marker genes identifying cell clusters in Supp Figure 1A. **(C)** Violin plots of cell-type specific marker genes identifying the cell clusters in Supp. Figure 1A. **(D)** UMAP of hypothalamic single cell RNA sequencing dataset. Raw data by Campbell et al. **(E)** Featureplots of cell-type specific marker genes identifying the cell clusters in Supp. Figure 1D. **(F)** Violin plots of cell-type specific marker genes identifying the cell clusters in Supp. Figure 1D. **(G)** UMAP of hypothalamic single nucleus RNA sequencing dataset. Raw data by Deng et al. **(H)** Featureplots of cell-type specific marker genes identifying the cell clusters in Supp. Figure 1G. **(I)** Violin plots of cell-type specific marker genes identifying the cell clusters in Supp. Figure 1G. **(J)** UMAP of hypothalamic single cell RNA sequencing dataset. Raw data by Chen et al. **(K)** Featureplots of cell-type specific marker genes identifying the cell clusters in Supp. Figure 1J. **(L)** Violin plots of cell-type specific marker genes identifying the cell clusters in Supp. Figure 1J.

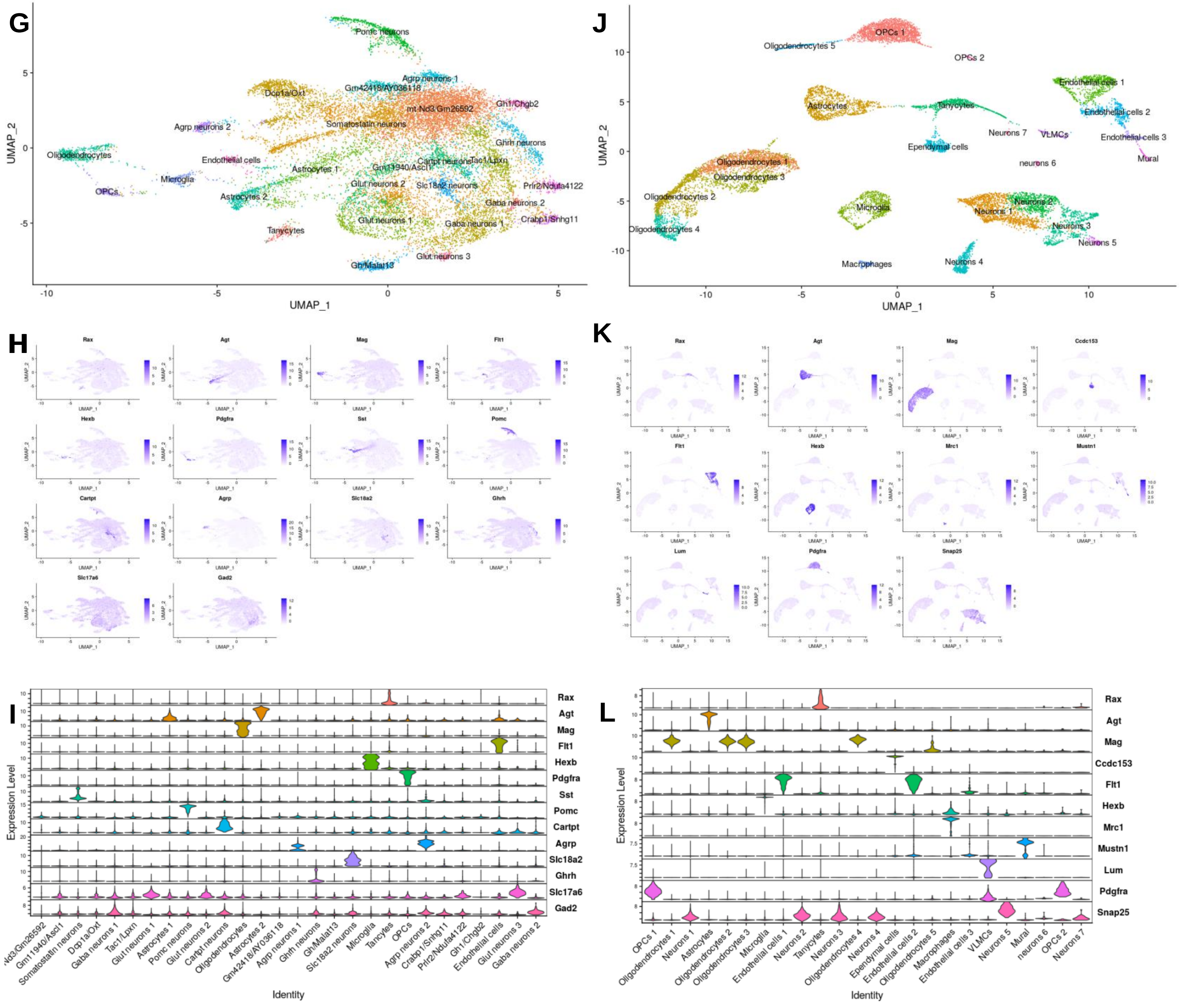
G
Pomc neurons
Agrp neurons 1
Gm42418/AY036118
Dcp1a/Oxt
mt-Nd3/Gm26592
Gh1/Chgb2
Somatostatin neurons
Agrp neurons 2
Ghrh neurons
Oligodendrocytes
Endothelial cells
Astrocytes 1
Cartpt neurons
Tac1/Lpxn
Gm11940/Ascl1
Microglia
Glut neurons 2
Slc18a2 neurons
Prlr2/Ndufa4122
OPCs
Astrocytes 2
Gaba neurons 2
Glut neurons 1
Gaba neurons 1
Crabp1/Snhg11
Tanycytes
Glut neurons 3
Gh/Malat13
UMAP_1
UMAP_2
J
OPCs 1
Oligodendrocytes 5
OPCs 2
Endothelial cells 1
Astrocytes
Tanycytes
Endothelial cells 2
Neurons 7
VLMCs
Endothelial cells 3
Ependymal cells
Mural
Oligodendrocytes 1
neurons 6
Oligodendrocytes 3
Oligodendrocytes 2
Microglia
Neurons 2
Neurons 1
Oligodendrocytes 4
Neurons 3
Neurons 5
Macrophages
Neurons 4
UMAP_1
UMAP_2
H
Rax
Agt
Mag
Flt1
Hexb
Pdgfra
Sst
Pomc
Cartpt
Agrp
Slc18a2
Ghrh
Slc17a6
Gad2
K
Rax
Agt
Mag
Ccdc153
Flt1
Hexb
Mrc1
Mustn1
Lum
Pdgfra
Snap25
I
Rax
Agt
Mag
Flt1
Hexb
Pdgfra
Sst
Pomc
Cartpt
Agrp
Slc18a2
Ghrh
Slc17a6
Gad2
Expression Level
Identity
L
Rax
Agt
Mag
Ccdc153
Flt1
Hexb
Mrc1
Mustn1
Lum
Pdgfra
Snap25
Expression Level
OPCs 1
Oligodendrocytes 1
Neurons 1
Astrocytes
Oligodendrocytes 2
Oligodendrocytes 3
Microglia
Endothelial cells 1
Neurons 2
Tanycytes
Neurons 3
Oligodendrocytes 4
Neurons 4
Ependymal cells
Endothelial cells 2
Oligodendrocytes 5
Macrophages
Endothelial cells 3
VLMCs
Neurons 5
Mural
neurons 6
OPCs 2
Neurons 7
Identity

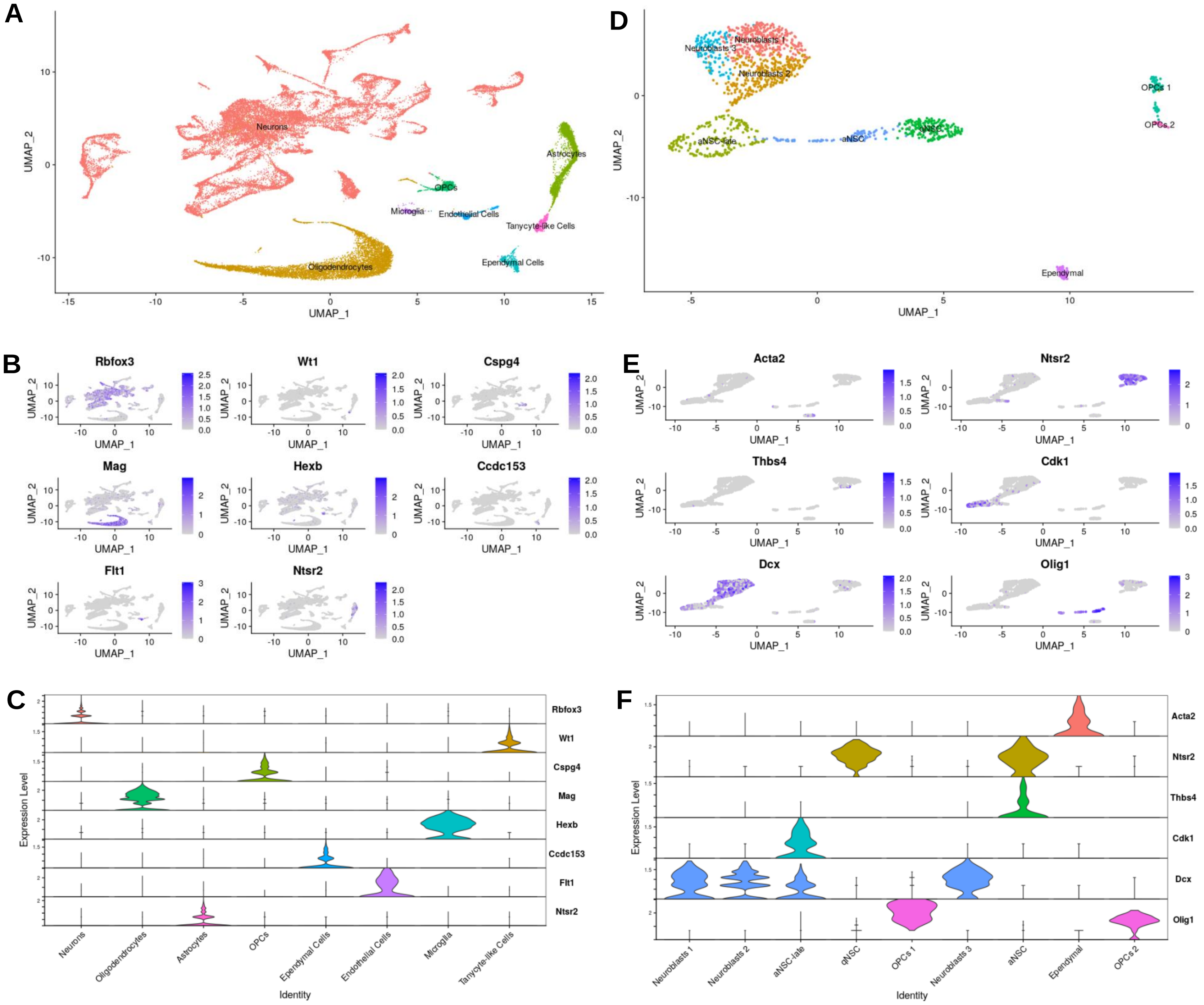

Figure S2: Identification of cell clusters in hindbrain and NSC reference datasets. **(A)** UMAP of combined hindbrain single cell RNA sequencing dataset. Raw data by Dowsett et al. and Ludwig et al. **(B)** Feature plots of cell-type specific marker genes identifying the cell clusters in Supp. Figure 2A. **(C)** Violin plots of cell-type specific marker genes identifying the cell clusters in Supp. Figure 2A. **(D)** UMAP of neural stem cell single cell RNA sequencing dataset. Raw data by Shah et al. **(E)** Featureplots of cell-type specific marker genes identifying the cell clusters in Supp Figure 2D. **(F)** Violin plots of cell-type specific marker genes identifying the cell clusters in Supp Figure 2D.

Table S1: Summary of datasets utilized in study. The following is a table containing the original publication title, author, gene expression omnibus (GEO) access code, sample type utilized from each dataset, the exact number of biological samples used from each condition, and the next generation sequencing platform used in each sample collection.

Publication	Author	Access	Sample Type	Number of Each Biological Samples Used	NGS platform
Single-Cell RNA-Seq Reveals Hypothalamic Cell Diversity	Renchao Chen	GSE87544	All	7 fasted, 10 chow	Illumina Hiseq 2500
Control of neurogenic competence in mammalian hypothalamic tanycytes	Sooyeon Yoo	GSE160378	scRNA-seq controls	3	Illumina NextSeq
A Molecular Census of Arcuate Hypothalamus and Median Eminence Cell Types	John N. Campbell	Lowell lab website	All but HFD	24 chow, 14 fast, 7 fast and re-fed, 3 low-fat diet	Illumina NextSeq 500
Single-Nucleus RNA Sequencing of the Hypothalamic Arcuate Nucleus of C57BL/6J Mice After Prolonged Diet-Induced Obesity	Guorui Deng	Direct access	Chow	7	Ilumina HiSeq 4000
Single-Cell Transcriptomics and Fate Mapping of Ependymal Cells Reveals an Absence of Neural Stem Cell Function	Prajay T. Shah	GSE100320	V-SVZ	5	Illumina HiSeq 2500
A survey of the mouse hindbrain in the fed and fasted state using single-nucleus RNA sequencing.	Georgina K C Dowsett	GSE168737	All	6 chow, 6 fast	Illumina NovaSeq 6000
A genetic map of the murine dorsal vagal complex and its role in obesity	Mette Q Ludwig	GSE166649	snRNA-seq vehicles	6	NextSeq 500 platform

Table S2: Software Versions. The following is a list of the version of each R software package utilized in this study.

R Studio 4.0.2	futile.logger 1.4.3	limma 3.46.0	Rhdf5lib 1.12.1	spatstat.geom 2.2-0	cli 3.1.0	gargle 1.2.0	lifecycle 1.0.1	quantmod 0.4.18	SQUAREM 2021.1
abind 1.4-5	futile.options 1.0.1	listenv 0.8.0	RhpcBLASctl 0.20-137	spatstat.linnet 2.2-1	clipr 0.7.1	generics 0.1.1	listenv 0.8.0	r2d3 0.2.5	stringi 1.7.5
batchelor 1.6.3	future 1.21.0	loomR 0.2.1.9000	Rhtslib 1.22.0	spatstat.sparse 2.0-0	colorspace 2.0-2	gert 1.4.1	lmtest 0.9-38	R6 2.5.1	stringr 1.4.0
beachmat 2.6.4	future.apply 1.7.0	lsa 0.73.2	ROCR 1.0-11	spatstat.utils 2.2-0	commonmark 1.7	ggplot2 3.3.5	lubridate 1.8.0	randomForest 4.6-14	sys 3.4
Biobase 2.50.0	gdata 2.18.0	Matrix.utils 0.9.8	rsample 0.1.0	spData 0.3.10	config 0.3.1	gh 1.3.0	magrittr 2.0.1	rappdirs 0.3.3	testthat 3.1.0
BiocGenerics 0.36.1	GenomeInfoDb 1.26.7	MatrixGenerics 1.2.1	Rsamtools 2.6.0	spdep 1.1-8	cpp11 0.4.0	gitcreds 0.1.1	memoise 2.0.0	rcmdcheck 1.4.0	tibble 3.1.5
BiocManager 1.30.16	GenomeInfoDbData 1.2.4	matrixStats 0.59.0	RSpectra 0.16-0	speedglm 0.3-3	crayon 1.4.1	globals 0.14.0	mime 0.12	RColorBrewer 1.1-2	tidyr 1.1.4
BiocNeighbors 1.8.2	GenomicRanges 1.42.0	miniUI 0.1.1.1	rsvd 1.0.5	stringr 1.4.0	credentials 1.3.1	glue 1.4.2	ModelMetrics 1.2.2.2	Rcpp 1.0.7	tidyselect 1.1.1
BiocParallel 1.24.1	ggforce 0.3.3	monocle3 1.0.0	Rtsne 0.15	SummarizedExperiment 1.20.0	curl 4.3.2	googledrive 2.0.0	modelr 0.1.8	RcppArmadillo 0.10.7.0.0	tidyverse 1.3.1
BiocSingular 1.6.0	ggfortify 0.4.12	parallelly 1.26.1	s2 1.0.6	tensor 1.5	data.table 1.14.2	googlesheets4 1.0.0	munsell 0.5.0	readr 2.0.2	timeDate 3043.102
BiocVersion 3.12.0	ggrepel 0.9.1	patchwork 1.1.1	S4Vectors 0.28.1	tweenr 1.0.2	DBI 1.1.1	gower 0.2.2	numDeriv 2016.8-1.1	readxl 1.3.1	tinytex 0.34
Biostrings 2.58.0	ggridges 0.5.3	pbapply 1.4-3	scattermore 0.7	units 0.7-2	dbplyr 2.1.1	gtable 0.3.0	nycflights13 1.0.2	recipes 0.1.17	tseries 0.10-48
bitops 1.0-7	ggseqlogo 0.1	pbmcapply 1.5.0	sctransform 0.3.2	uwot 0.1.10	desc 1.4.0	haven 2.4.3	openssl 1.4.5	rematch 1.0.1	TTR 0.24.2
caTools 1.18.2	glmGamPoi 1.2.0	pheatmap 1.0.12	scuttle 1.0.4	viridis 0.6.1	devtools 2.4.2	hexbin 1.28.2	parallelly 1.28.1	rematch2 2.1.2	tzdb 0.2.0
classInt 0.4-3	gmodels 2.18.1	plotly 4.9.4.1	Seurat 4.0.3	warp 0.2.0	diffobj 0.3.5	highr 0.9	pbdZMQ 0.3-5	remotes 2.4.1	urca 1.3-0
coda 0.19-4	goftest 1.2-2	png 0.1-7	SeuratDisk 0.0.0.9019	wk 0.4.1	digest 0.6.28	hms 1.1.1	pillar 1.6.4	repr 1.1.3	usethis 2.1.3
cowplot 1.1.1	gplots 3.1.1	polyclip 1.10-0	SeuratObject 4.0.2	XVector 0.30.0	dplyr 1.0.7	htmltools 0.5.2	pkgbuild 1.2.0	reprex 2.0.1	utf8 1.2.2
crosstalk 1.1.1	gridExtra 2.3	proxy 0.4-26	SeuratWrappers 0.3.0	zlibbioc 1.36.0	dtplyr 1.1.0	htmlwidgets 1.5.4	pkgconfig 2.0.3	reshape2 1.4.4	uuid 1.0-2
DelayedArray 0.16.3	grr 0.9.5	pscl 1.5.5	sf 1.0-1	askpass 1.1	e1071 1.7-9	httpuv 1.6.3	pkgload 1.2.3	rlang 0.4.12	vctrs 0.3.8
DelayedMatrixStats 1.12.3	gtools 3.9.2	qlcMatrix 0.9.7	Signac 1.2.1	assertthat 0.2.1	ellipsis 0.3.2	httr 1.4.2	plogr 0.2.0	rmarkdown 2.11	viridisLite 0.4.0
deldir 0.2-10	harmony 0.1.0	RANN 2.6.1	SingleCellExperiment 1.12.0	backports 1.3.0	evaluate 0.14	ids 1.0.1	plyr 1.8.6	roxygen2 7.1.2	vroom 1.5.5
devtools 2.4.2	HDF5Array 1.18.1	raster 3.4-13	sitmo 2.0.1	base64enc 0.1-3	fansi 0.5.0	ini 0.3.1	praise 1.0.0	rprojroot 2.0.2	waldo 0.3.1
docopt 0.7.1	hdf5r 1.3.3	RcppAnnoy 0.0.18	slam 0.1-48	bit 4.0.4	farver 2.1.0	ipred 0.9-12	prettyunits 1.1.1	RSQLite 2.2.8	whisker 0.4
dqrng 0.3.0	ica 1.0-2	RcppEigen 0.3.3.9.1	slider 0.2.2	bit64 4.0.5	fastmap 1.1.0	IRdisplay 1.0	pROC 1.18.0	rstudioapi 0.13	withr 2.4.2
DropSeq.util 2.0	igraph 1.2.6	RcppHNSW 0.3.0	snakecase 0.11.0	blob 1.2.2	fontawesome 0.2.2	IRkernel 1.2	processx 3.5.2	rversions 2.1.1	xfun 0.27
e1071 1.7-7	IRanges 2.24.1	RcppProgress 0.4.2	snow 0.4-3	brew 1.0-6	forcats 0.5.1	isoband 0.2.5	prodlim 2019.11.13	rvest 1.0.2	xml2 1.3.2
expm 0.999-6	irlba 2.3.3	RcppRoll 0.3.0	SnowballC 0.7.0	brio 1.1.2	foreach 1.5.1	iterators 1.0.13	progress 1.2.2	sass 0.4.0	xopen 1.0.0
fansi 0.5.0	janitor 2.1.0	RCurl 1.98-1.3	sp 1.4-5	broom 0.7.9	forecast 8.15	jquerylib 0.1.4	progressr 0.9.0	scales 1.1.1	xtable 1.8-4
fastmatch 1.1-0	lambda.r 1.2.4	remotes 2.4.0	sparseMatrixStats 1.2.1	bslib 0.3.1	forge 0.2.0	jsonlite 1.7.2	promises 1.2.0.1	selectr 0.4-2	xts 0.12.1
fitdistrplus 1.1-5	lazyeval 0.2.2	ResidualMatrix 1.0.0	sparsesvd 0.2	cachem 1.0.6	fracdiff 1.5-1	knitr 1.36	proxy 0.4-26	sessioninfo 1.1.1	yaml 2.2.1
FNN 1.1.3	LearnBayes 2.15.1	reticulate 1.20	spatstat 2.2-0	callr 3.7.0	fs 1.5.0	labeling 0.4.2	ps 1.6.0	shiny 1.7.1	zip 2.2.0
formatR 1.11	leiden 0.3.8	rhdf5 2.34.0	spatstat.core 2.2-0	caret 6.0-90	future 1.22.1	later 1.3.0	purrr 0.3.4	sourcetools 0.1.7	zoo 1.8-9
furrr 0.2.3	leidenbase 0.1.3	rhdf5filters 1.2.1	spatstat.data 2.1-0	cellranger 1.1.0	future.apply 1.8.1	lava 1.6.10	quadprog 1.5-8	sparklyr 1.7.2	

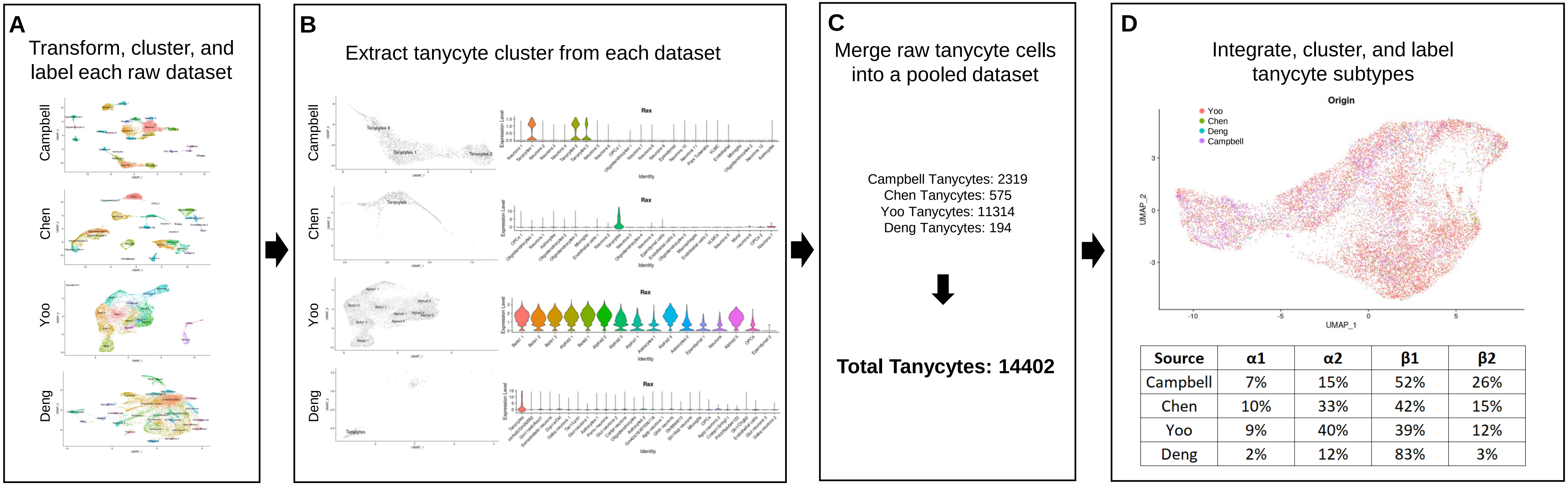

Source	α1	α2	β1	β2
Campbell	7%	15%	52%	26%
Chen	10%	33%	42%	15%
Yoo	9%	40%	39%	12%
Deng	2%	12%	83%	3%

Figure S3: Workflow of tanycyte isolation and integration. **(A)** First, each raw dataset was transformed, clustered, and labeled to allow for extraction of tanycytes (specific functions utilized to achieve this can be found in the methods). **(B)** Tanycyte populations from each dataset were isolated. Clusters “Tanycytes 1,” “Tanycytes 2,” and “Tanycytes 3” were isolated from the Campbell dataset. Cluster “Tanycytes” was isolated from the Chen dataset. Clusters “Alpha1 1,” ”Alpha2 1,” ”Alpha2 2,” ”Alpha2 3,” ”Alpha2 4,” ”Alpha2 5,” ”Beta1 1,” ”Beta1 2,” ”Beta1 3,” ”Beta2 1” were isolated from the Yoo dataset. Cluster ”Tanycytes” was isolated from the Deng dataset. **(C)** The raw cells from each dataset’s isolated tanycyte clusters were then merged into one dataset. **(D)** This merged dataset was then integrated by origin, clustered, and finally labeled by tanycyte subtype. The table contains the percent of each dataset that clustered as each tanycyte subtype.

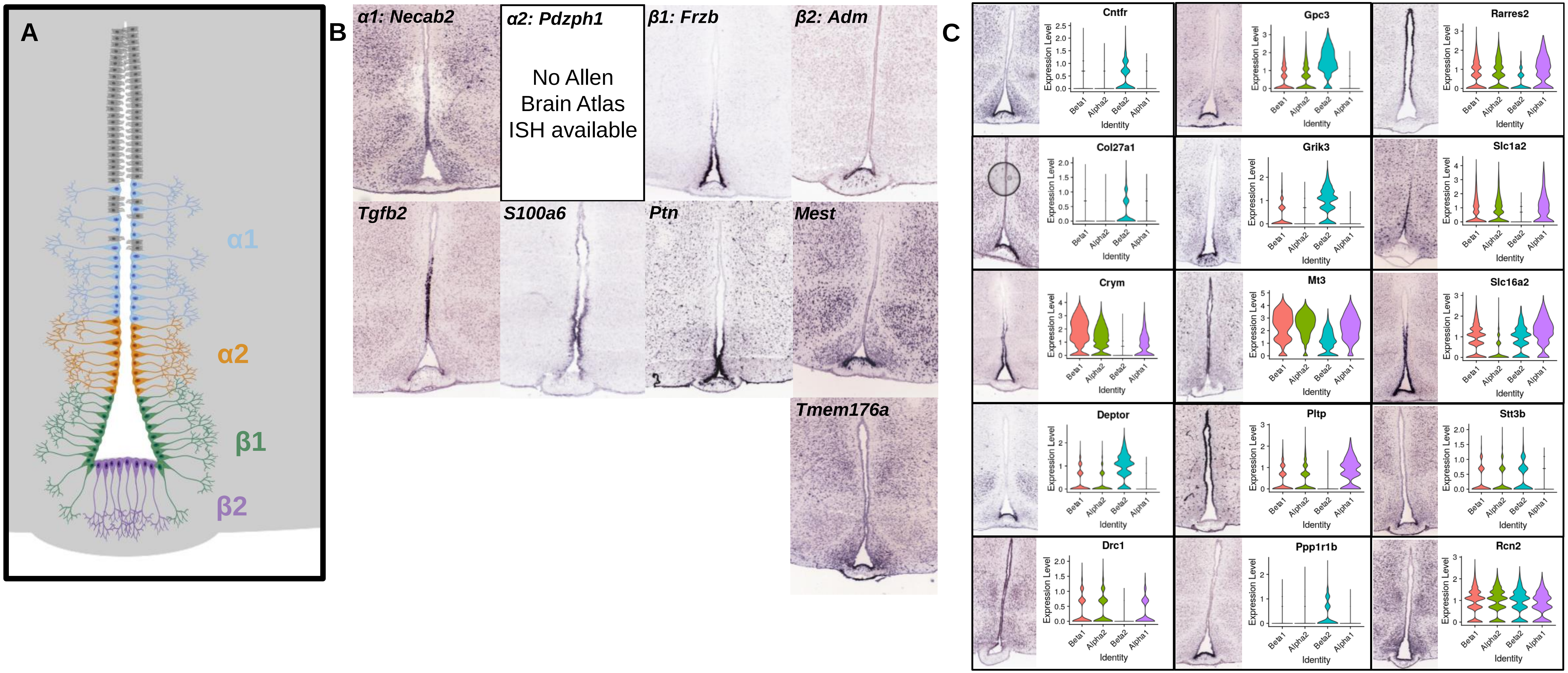

Figure S4: Allen brain atlas ISH secondary verification of tanycyte subtype classification. **(A)** Schematic representation of the tanycyte subtypes. **(B)** In situ hybridization (ISH) of subtype marker transcripts (top row) and of sample top ten transcripts upregulated in each subtype cluster (other rows). Only Allen brain atlas ISH deposits that were coronal, passed quality control parameters, and showed expression in the third ventricle were included. **(C)** ISH of transcripts from the Allen brain atlas third ventricle fine structure atlas. 41 of the genes in this atlas showed uniform expression across all tanycyte subtypes. The 15 genes that did not show uniform ISH expression in all tanycytes are shown above with accompanying violin plots showing their expression in the combined tanycyte dataset.
